# Processing Stabilization of Polyethylene with Grape Peel Extract: Effect of Extraction Technology and Composition

**DOI:** 10.3390/molecules28031011

**Published:** 2023-01-19

**Authors:** Kata Takács, Emese Pregi, Erika Vági, Tibor Renkecz, Dóra Tátraaljai, Béla Pukánszky

**Affiliations:** 1 Institute of Materials and Environmental Chemistry, Research Centre for Natural Sciences, Magyar Tudósok Körútja 2, 1117 Budapest, Hungary; 2 Laboratory of Plastics and Rubber Technology, Department of Physical Chemistry and Materials Science, Faculty of Chemical Technology and Biotechnology, Budapest University of Technology and Economics, Műegyetem rkp. 3, 1111 Budapest, Hungary; 3 Department of Chemical and Environmental Process Engineering, Faculty of Chemical Technology and Biotechnology, Budapest University of Technology and Economics, Műegyetem rkp. 3, 1111 Budapest, Hungary; 4 Toxi-Coop Toxicological Research Center, Berlini u. 47-49, 1045 Budapest, Hungary

**Keywords:** grape peel extract, polyphenols, DPPH assay, inherent stability, melt stabilization, long-term stability, color

## Abstract

Dry grape peel powder was extracted by three different techniques, stirred tank reactor, Soxhlet and ultrasound extraction. The composition, physical and chemical structure and inherent stability of the extracts were characterized by various methods. The extracts and reference compounds were added to polyethylene and their stabilization efficiency was determined in multiple extrusion experiments. The composition of the extracts was quite similar. Ten main compounds were identified in the extracts, which contained a considerable number of polyphenols, but only small amounts of quercetin and *trans*-resveratrol. The extracts proved to be more efficient processing stabilizers than *trans*-resveratrol and the commercial stabilizer, Irganox 1010, irrespective of the extraction technology used. In spite of their good processing stabilization effect, polymers containing the extracts had poor residual stability. The differences in processing and long-term stabilization must be related to the different structures of the polyphenols contained by the extracts and the reference compounds. The results clearly prove that the IC50 value determined by the DPPH assay is not suitable for the estimation of the efficiency of a compound as a stabilizer for polymers.

## 1. Introduction

The majority of polymer products are produced from thermoplastics by melt processing technologies. They are subjected to elevated temperatures and shear during conversion. Chemical reactions take place during processing, changing the structure of the polymer practically always [1,2]. Accordingly, polymers must be protected against these changes to ensure safe processing and conserve useful end properties. The stabilization of polymers is a well-established technology, during which a hindered phenolic antioxidant and a phosphorous or a sulfur-containing secondary antioxidant are usually added to protect the polymer against thermo-oxidative degradation [1,3,4]. The approach is widely used for the stabilization of polyolefins, including polyethylene as well. The currently used additive packages are very efficient, but environmental concerns [5] and the general drive towards the use of raw materials from natural origins have increased the interest in natural antioxidants [6].

Nature produces and uses a huge number of compounds with antioxidant activity [7,8]. Many of these are already applied in the food industry, and several have been tried as stabilizers in polymers. Lignin, for example, has some stabilization effect, but the relatively small molar concentration of phenolic hydroxyl groups, its insolubility in polymers and its strong color prevent its widespread use as a stabilizer in practice [9,10,11]. The first natural antioxidant routinely used in practice was α-tocopherol or vitamin E, which is added to medical devices to ensure long-term stability [12,13,14]. However, the number of studies and, thus, scientific papers on the use of natural antioxidants for the stabilization of plastic products continuously increases. Flavonoids produced by plants, such as quercetin [15,16,17], rutin [18] and dihydromyricetin [19], proved efficient stabilizers in polyethylene, and the comparison of a natural extract, silymarin, to its main component, silybin, showed that extracts could be more efficient than purified single compounds [20]. Recently, pomegranate peel extract was used for the processing stabilization of polyethylene, and its efficiency exceeded that of the commercial hindered phenolic antioxidant, Irganox 1010 (I1010) [21,22]. Unfortunately, the residual, long-term stability of the polyethylene containing the extract was quite small; thus, further study is needed to find an optimum solution for the use of this extract.

Winery by-products are rich in bioactive molecules, such as phenolic acids, flavonoids, anthocyanins, *trans*-resveratrol and the glycosides of these substances [23]. Grape skins represent 5–10% of the total dry weight of the grape berry. The wine industry annually produces 5–7 million tons of grape pomace resulting from the processing of 43 million tons of grapes [24]. The phenolic content of grape skin ranges from 285–550 mg phenols/kg grape skin, depending on the grape variety and the type of pre-treatment [25]. Not only the number of phenolic compounds but also their composition varies widely with the type of grape, climatic conditions, and extraction technology [26,27,28]. Flavonoids are assumed to be the most efficient antioxidant components in grape extracts, but stilbenes and phenolic acids also may contribute to stabilization. Most sources agree that *trans*-resveratrol and quercetin are present in the various parts of the grape, but contradictory information has been published about their number and location. Du et al. [29], for example, followed changes in the number of various compounds with antioxidant activity in the peel of grapes during the growth of the plant and found that the quantity of practically all components increases with ripening. On the other hand, Jeandet et al. [30] claim that grape peel does not contain much resveratrol, and its amount decreases during growth. Some of the studies also indicate that most flavonoids are present in grape peel in the form of glycosides, which might hinder their application as processing stabilizers because of the limited inherent stability of such derivatives [8,18].

The stabilization efficiency of various winery by-products and compounds has already been studied in different polymers. Cerruti et al. [31,32] stabilized polypropylene (PP) with a number of extracts and grape seed powders. The additive was obtained from Cabernet pomace through hydroalcoholic extraction at room temperature. The results revealed that the efficiency of the extract at 5000 ppm is comparable to an industrial antioxidant at 1000 ppm, but the homogeneity of the samples was not satisfactory. Nanni et al. [33] incorporated three different solid wine wastes (peels, seeds and stalks) into PP and tested them as stabilizers. Their stabilizing activity was compared with that of a commercial tannin extract powder rich in polyphenols. The stabilizers were added to the polymer at 6 wt%. The wine wastes increased the oxidation induction time (OIT) and the oxidation onset temperature (OOT) of the polymer and did not influence the mechanical properties of the samples, but homogeneity was a problem also in this case. Similarly, biopolymers, such as poly(3-hydroxybutyrate) (PHB) or Materi-Bi^®^, have been stabilized using antioxidants derived from the winery industry [34,35]. Natural extracts from grape pomace and grape seed were used for the stabilization of poly(butylene succinate) (PBS) as well [36]. The commercial seed extract was found to be a very efficient stabilizer showing results comparable to, if not even better than, Irganox 1010. Not only various winery by-products and extracts but also some of their vital components have been tried as stabilizers in polymers. Resveratrol, for example, was added to polyethylene films used in food packaging applications alone or with some supporting material (starch, montmorillonite), resulting in improved stability and antimicrobial activity, and thus improved shelf life for the food [37]. Winery by-products obviously are potential additives for polymers, but the available information is scarce and contradictory; thus, further study is needed in this area.

Accordingly, the goal of this study was to prepare extracts from grape peel available in considerable quantities in Hungary and investigate their possible use as stabilizers in polyethylene. Three different technologies were used for extraction to determine their effect on the composition and antioxidant activity of the extracts. Composition and activity were characterized by various means, and then the extracts were added to a Phillips-type high-density polyethylene to study their effect on the properties of the polymer. The commercial stabilizer used in the largest quantity in industrial practice (I1010) and *trans*-resveratrol were used as reference stabilizers. All additive packages contained a phosphorous secondary stabilizer as well to comply with industrial practice. Changes in the structure and properties of the polymer during multiple extrusions were followed by various techniques.

## 2. Results

The results are presented in several sections. The extraction technology, its results and the composition of the obtained extracts are discussed first, and then the extracts are characterized by various techniques. The stabilization efficiency of the three extracts is compared to that of the commercial stabilizer, I1010 and *trans*-resveratrol, in the next section, while correlations and practical relevance are mentioned in the final part of the paper.

### 2.1. Extraction Technology and Composition

**Figure 1:**
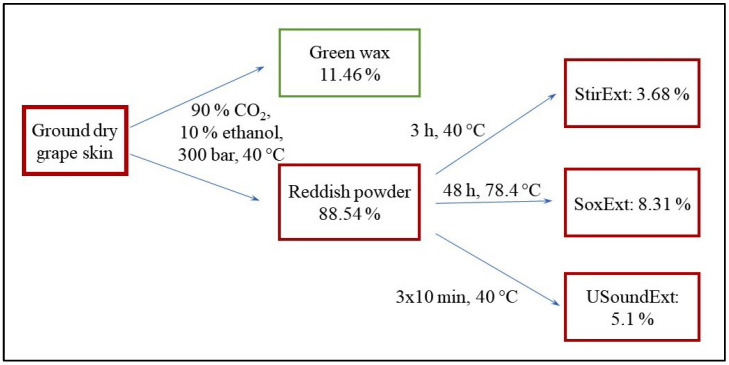
Schematic presentation of the extraction process, conditions and yield.

The scheme of the extraction procedure is shown in Figure 1. Ground dry grape peel powder was first extracted with supercritical fluid extraction using a mixture of 90% CO_2_ and 10% ethanol at 40 °C and 300 bar. A green waxy material (11.46 wt%) and a reddish powder (88.54 wt%) were obtained in the procedure. The first was tested for polyphenols (<1 wt%) and antioxidant activity (250 µg/mL; DPPH assay), but both tests were largely negative; thus, the component was discarded. The reddish residue was further extracted by three different methods: stirring in a tank reactor (Stir), Soxhlet (Sox) and ultrasound extraction (USound). The extraction conditions and yield are shown in Figure 1, and the latter is also in Table 1. About 5 wt% extract was obtained in the average related to the reddish powder obtained in the first step. The three extracts were analyzed by various techniques to determine their composition and characteristics.

**Figure 2:**
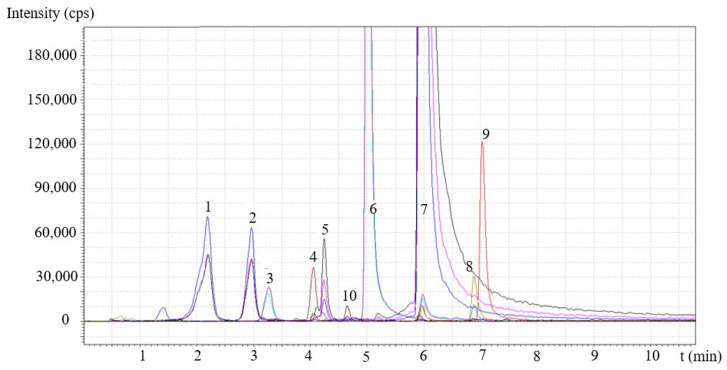
LC-MS/MS analysis of the composition of grape peel extract obtained by Soxhlet extraction. Different colors indicate different MRM transitions of the main components: (1) catechin, (2) epicatechin, (3) myricetin glycoside, (4) epicatechin gallate, (5) quercetin glycoside, (6) myricetin, (7) quercetin, (8) kaempferol, (9) isorhamnetin and (10) *trans*-resveratrol.

The presence of some main components and the number of selected compounds with large antioxidant activity (*trans*-resveratrol, quercetin) were determined by LC-MS/MS. A representative chromatogram obtained on the Stir extract is presented in Figure 2. The chromatogram was also magnified to show minor components. Altogether, 10 components were identified with certainty in the chromatogram in smaller or larger amounts. The amount of *trans*-resveratrol and quercetin was quantified using standards, while some of the other components were identified using information (MRM transitions) published in the literature [23]. It is worth mentioning that a wide variety of other compounds must be present, but in this study, we did not aim to give an in-depth component profile of the extracts. The compounds found are listed in the caption of Figure 2. The yield of the three extraction techniques and the composition of the extracts are collected in Table 1. The composition of the three extracts was very similar; only the relative amount of the components differed somewhat from one technique to the other.

According to Table 1, all three extracts contain considerable amounts of polyphenols, their content being between 10 and 20 wt% of the respective extracts. What is surprising, however, is the very small amount of quercetin and the even smaller quantity of *trans*-resveratrol found in the extracts. Grape peel extract is expected to contain larger amounts of *trans*-resveratrol, which is supposed to account for its strong antioxidant effect [38,39,40]. The small amounts might have several reasons. The extracts of the red grape peel obtained from the Gere vinery in Villány, Hungary, might not have contained considerable amounts of the compound. *Trans*-resveratrol might have been extracted in the first step of the procedure and discarded, but the results of the polyphenol and DPPH tests contradict this possibility. Finally, both *trans*-resveratrol and quercetin can be present as glycosides, and the method used could not quantify them based on the available standards. Several sources claim that the majority of the flavonoids in the grape peel are present in the form of glycosides [23,29,41]. Nevertheless, further study of the extraction procedure and the composition of the extracts are needed to answer these questions satisfactorily.

### 2.2. Characterization of the Extracts

Several factors determine the effect and efficiency of compounds as a stabilizer, including their chemical and physical structure, melting point if they are crystalline, inherent stability, solubility in the matrix polymer, etc. [1]. These aspects of the extracts were investigated by a number of methods. The possible stabilization effect of the extracts was estimated by the DPPH assay. DPPH reacts with compounds forming free radicals by the splitting off of their active hydrogen atoms. The results are reported as IC50 values indicating the concentration of a compound causing a 50% loss in DPPH activity. A smaller IC50 value means larger antioxidant activity. The results of the DPPH test on the three extracts and the compounds used as references are listed in Table 2. Quercetin possesses the smallest IC50 value, and it was shown earlier that this compound is a very efficient stabilizer in polyethylene [15]. The largest IC50 value obtained was for the commercial hindered phenolic antioxidant, Irganox 1010, which is somewhat surprising. The three extracts have similar IC50 values as *trans*-resveratrol, which is known to be a very efficient antioxidant [38]. Accordingly, we may expect the extracts to perform well as stabilizers in PE. Table 2 also lists the polyphenol content of the extracts, which seems to correlate to some extent with their antioxidant activity. Consequently, we may expect better stabilization efficiency from extracts produced by the stirred tank and Soxhlet extractions than from the ones prepared by ultrasound extraction.

**Figure 3:**
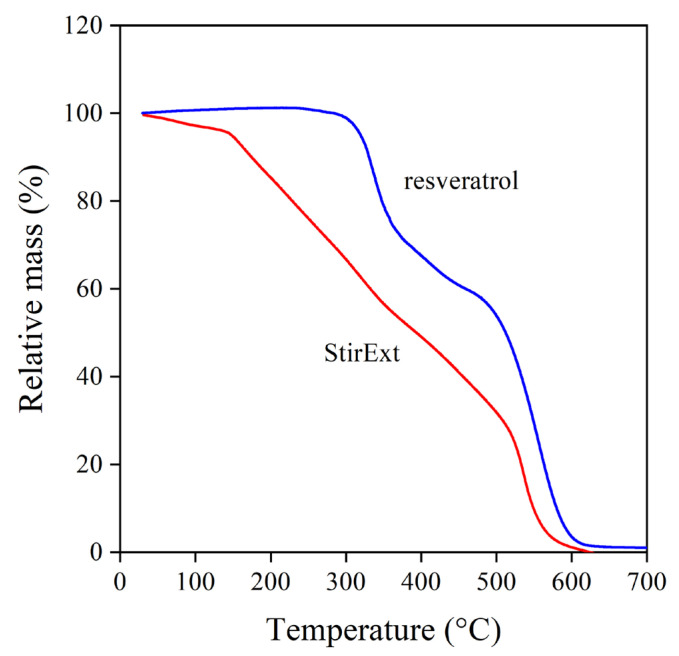
Thermogravimetric analysis of *trans*-resveratrol and grape peel extract produced in the stirred tank reactor.

The inherent stability of a compound also influences its possible use and efficiency as a stabilizer. Rutin, the glycoside derivative flavonoid, was inferior to quercetin because of the decomposition of the glycoside moiety [18]. The decomposition of the extracts and the reference compounds was studied by thermogravimetric analysis (TGA). The thermogram of one of the extracts (StirExt) and that of *trans*-resveratrol are shown in Figure 3. The behavior of the other two extracts was practically identical to that shown. The decomposition of the two materials drastically differs. *Trans*-resveratrol is stable up to about 300 °C and then decomposes in two steps. The grape extract, on the other hand, already starts to decompose at very low temperatures, and considerable weight loss occurs already below temperatures occurring during the processing of polyethylene. The limited stability corroborates the assumption that glycoside derivatives are present in large quantities in the extract, on the one hand, and forecasts problems using it as a stabilizer on the other.

**Figure 4:**
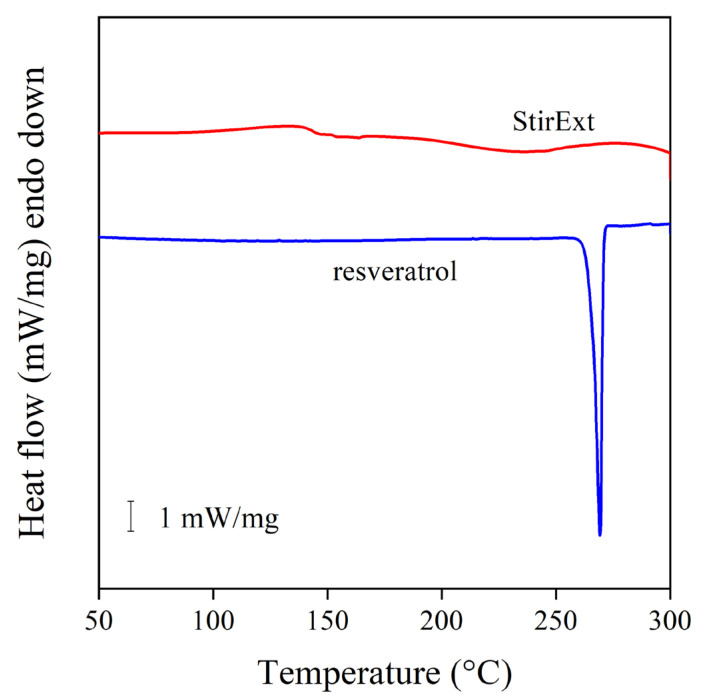
Comparison of the DSC thermogram of *trans*-resveratrol and the extract obtained by stirred extraction. Heating at 10 °C/min rate in oxygen.

Another issue, which must be considered in the use of a compound as a stabilizer for polymers, is its physical form and melting characteristics. Quercetin is a crystalline compound with a very high melting temperature (320 °C), and thus it does not melt during processing, which creates homogenization problems [15]. The DSC traces of an extract and the reference compound, *trans*-resveratrol, are presented in Figure 4. The trace of the latter clearly shows that the compound is crystalline with a high melting point (270 °C), while the extract is completely amorphous. Accordingly, one could expect better solubility and homogeneity in the case of the extracts than with the pure compounds, either *trans*-resveratrol or quercetin.

**Figure 5:**
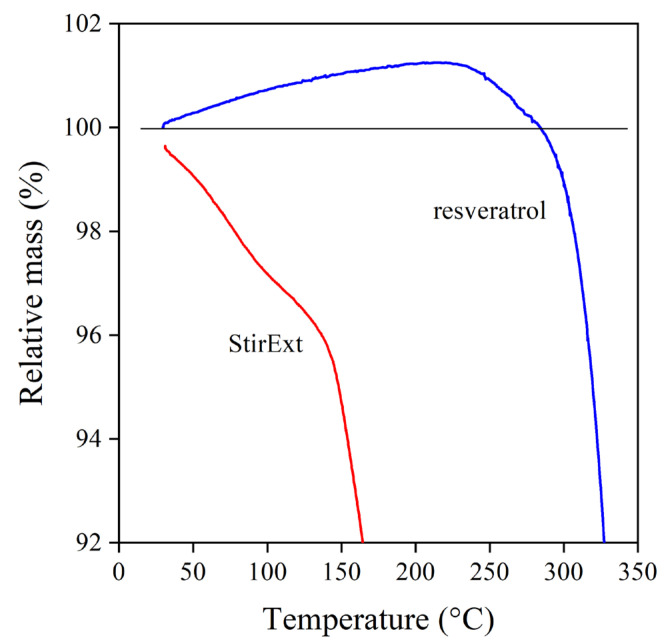
TGA traces of *trans*-resveratrol and the grape peel extract were produced in the stirred tank reactor. O_2_ atmosphere; reaction of *trans*-resveratrol with oxygen.

Finally, the thermal analysis offers further information on the possible behavior of the various compounds as stabilizers. The TGA trace of *trans*-resveratrol and one of the extracts (StirExt) is presented in Figure 5 at lower temperatures than in Figure 3, below 350 °C. According to the figure, the extract starts to decompose at very low temperatures, already at the beginning of the test indeed. On the other hand, the weight of *trans*-resveratrol increases up to about 220 °C, indicating its reaction with molecular oxygen. Since adsorbed and dissolved oxygen present in PE during processing results in hydrogen abstraction, radical formation, and degradation, scavenging oxygen by any compound is very beneficial, resulting in efficient melt stabilization. The characterization of the extracts and the reference compounds indicates that the latter should be good stabilizers for polymers, while stabilization efficiency is doubtful in the case of the extracts.

### 2.3. Stabilization Efficiency

**Figure 6:**
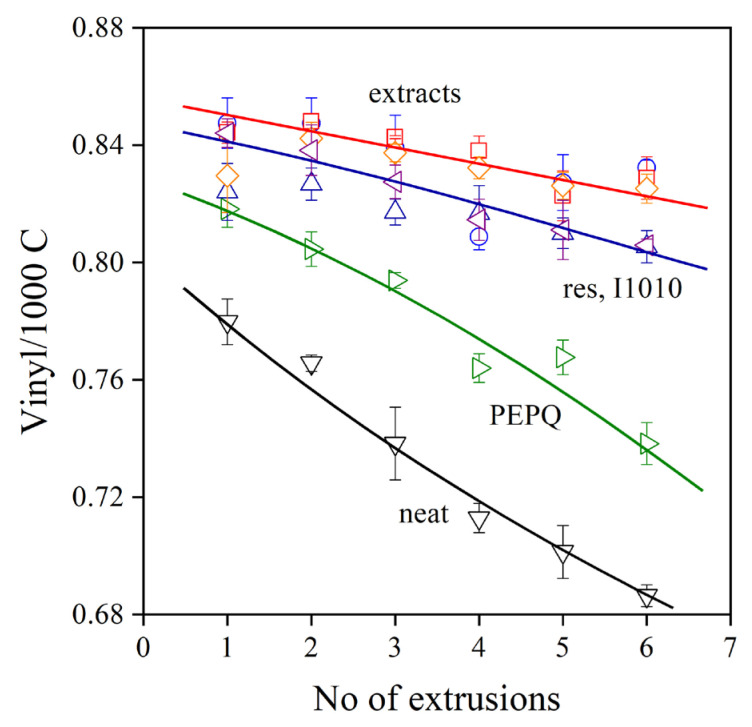
Vinyl group content of polyethylene containing various additive packages plotted against the number of extrusion steps. Symbols: (▽) neat polymer, (▷) PEPQ, (△) I1010, (◁) resveratrol, (☐) StirExt, (◯) SoxExt and (◇) UsoundExt. Additive content: 1000 ppm PEPQ was added to packages containing 1000 ppm phenolic antioxidants.

The Phillips-type polyethylene used in this study has a double bond at the end of each chain, which takes part in reactions during processing and use. The addition of radicals to the vinyl groups leads to long-chain branching and an increase of viscosity, and thus a decrease of MFR. Accordingly, changes in the vinyl content during processing are indications of the number of reactions taking place, and in the presence of additives, it shows the efficiency of the stabilizer in preventing degradation. The vinyl group content of PE used in this study is plotted against the number of subsequent extrusions in Figure 6. All the references, the neat polymer, the PE processed only in the presence of the secondary stabilizer, the polymer processed with *trans*-resveratrol and the commercial stabilizer, I1010, are also plotted in the figure. The vinyl content of the neat polymer drastically decreases in the first but also in subsequent extrusions, and considerable degradation takes place. PEPQ, the phosphorous secondary stabilizer, offers some protection for the polymer, but it is rapidly consumed, as shown by the relatively fast decrease in the vinyl content of the polymer. *Trans*-resveratrol and I1010 stabilize the polymer, but rather surprisingly, the smallest decrease in vinyl content occurs in the presence of the three extracts. All three seem to be very efficient processing stabilizers; their effect is very similar, and differences in their efficiency cannot be observed practically at all.

**Figure 7:**
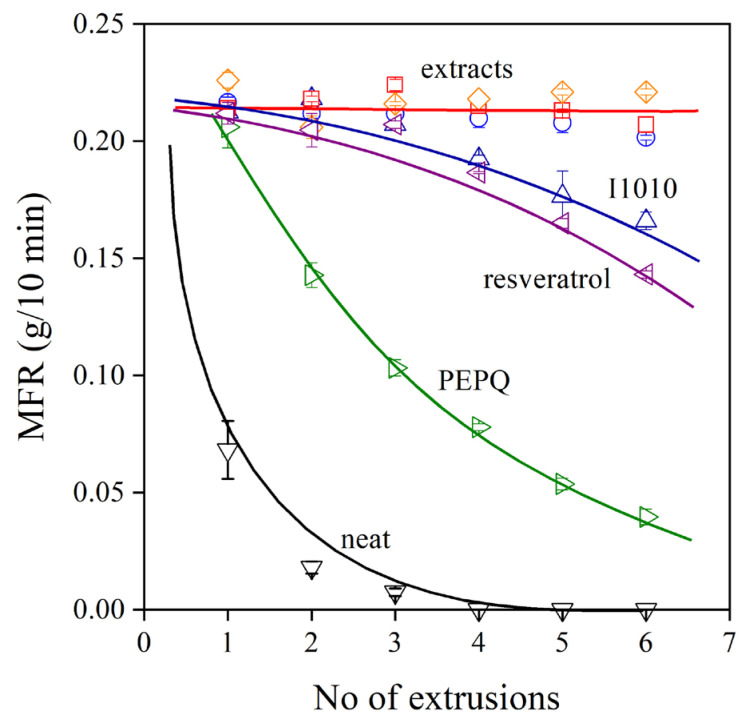
Effect of processing history (number of extrusions) on the viscosity (melt flow rate) of polyethylene processed in the presence of various additive packages. Symbols are the same as in Figure 6.

The melt flow rate (MFR) of the polymer containing the various additive packages is shown as a function of the number of extrusions in Figure 7. The dependence of viscosity on processing history completely corroborates the results shown in Figure 6, i.e., the changes in vinyl content are closely related to MFR. The MFR of the neat polymer rapidly decreases to very small values with the increasing number of extrusions, while that of the PE compounds containing both a phenolic and a secondary antioxidant changes much less. The three extracts produced from the grape peel are extremely efficient melt stabilizers, better than *trans*-resveratrol and I1010, and their effect is very similar.

**Figure 8:**
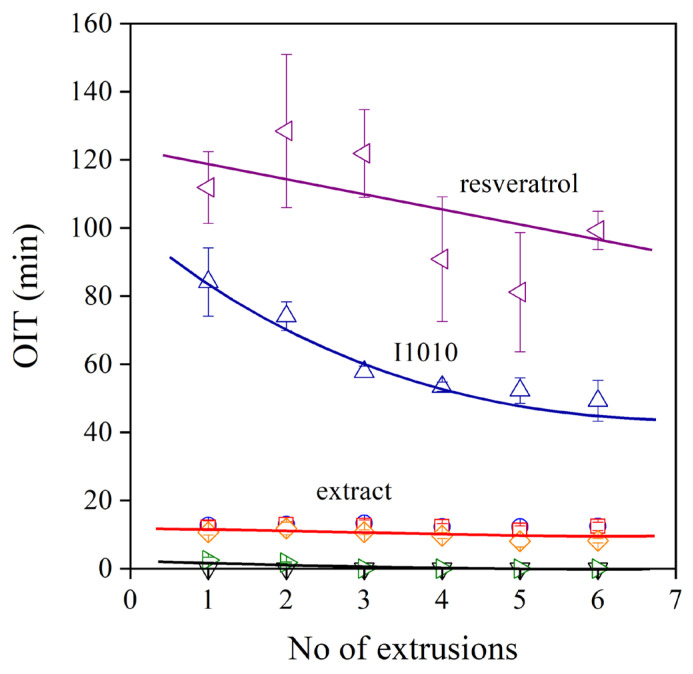
Dependence of the residual stability of HDPE stabilized with different phenolic compounds. Symbols are the same as in Figure 6.

An important issue in stabilization is the residual stability of the polymer after processing. Some applications, such as packaging films, do not require long-term stability, but others, such as pipes, do. Residual stability can be characterized by the oxidation induction time (OIT), and the obtained results on the prepared polyethylenes are plotted in Figure 8 against the number of extrusions. The neat polymer and the one containing only the secondary stabilizer (PEPQ) do not have any residual stability at all. On the other hand, *trans*-resveratrol is a very efficient stabilizer; its effect is much stronger than that of the commercial hindered phenol stabilizer (I1010). Unfortunately, the extracts have a very moderate stabilization effect; their OIT value hardly exceeds 10 min. The phenomenon has been observed before; the extract of pomegranate peel had a very good processing stabilization effect but offered no residual stability for the polymer [21,22].

**Figure 9:**
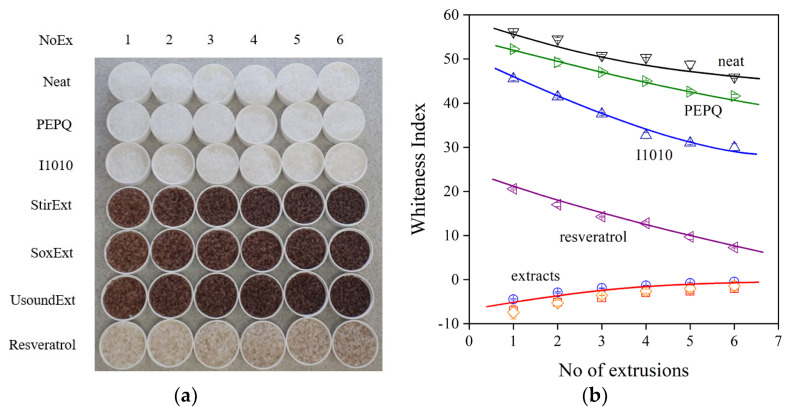
Discoloration of polyethylene containing various additive packages during processing. (**a**) A visual view of the samples and (**b**) whiteness index plotted against the number of extrusions. Symbols are the same as in Figure 6.

Another important question for some applications is the color of the product. A very small discoloration is allowed, for example, in most packaging applications. The drawback of natural antioxidants and extracts is that they have a strong inherent color, or the metabolites formed during stabilization discolor the polymer [6]. The strong color of the polymer containing the three extracts and *trans*-resveratrol is visually shown in Figure 9a. Color can be characterized by various quantities, such as the yellowness index, whiteness index or the optical L* parameter. The whiteness index determined for the polymers prepared with the various additive packages is presented in Figure 9b. The neat polymer and the one only containing PEPQ are quite white, and their color does not change much during multiple extrusions. The quinoidal compounds formed from I1010 give a slight yellowish-reddish tint to the polymer [42], while discoloration is very strong during the extrusion of the polymer in the presence of *trans*-resveratrol and especially with the extracts. Although the latter are very efficient processing stabilizers, their application might be hindered by the poor residual stability and the strong color of the polymer containing them.

## 3. Discussion

**Figure 10:**
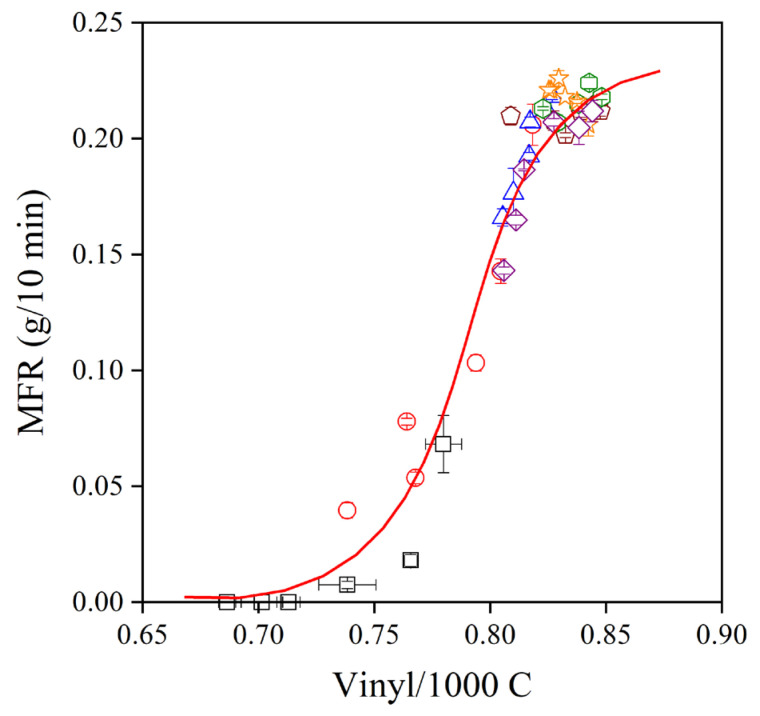
Correlation between the vinyl group content and melt flow rate of polyethylene containing different additive packages. Symbols are the same as in Figure 6.

**Figure 11:**
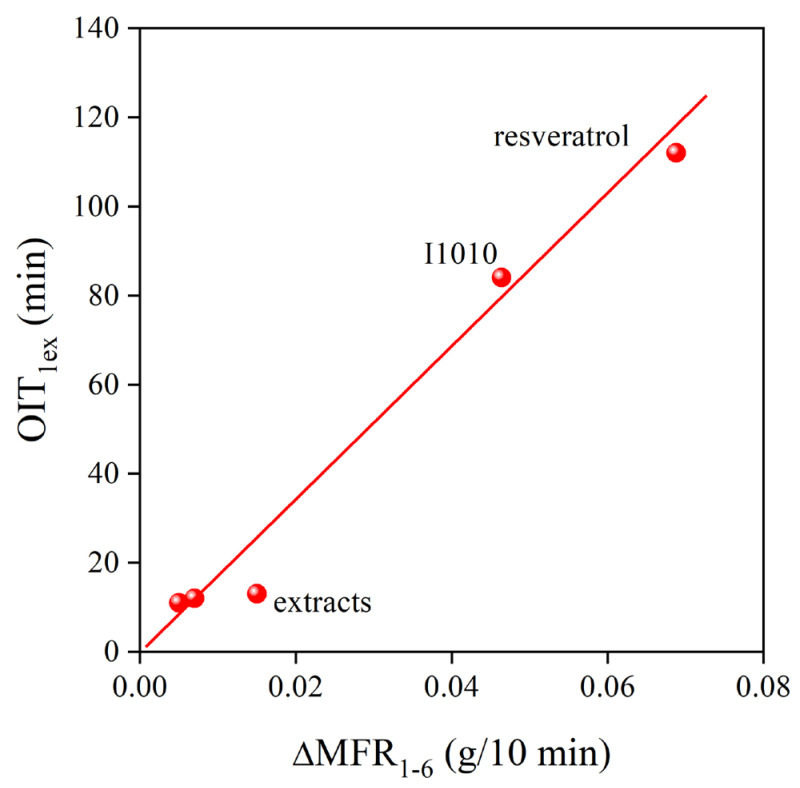
Inverse correlation between the residual and the processing stability of PE stabilized with the reference compounds and the grape peel extracts obtained by different techniques.

The very strong stabilization effect of natural antioxidants in polymers has been shown before [6]. However, their potential application is yet in its infancy, and intensive research is needed to explore their advantages and drawbacks. In the Phillips polyethylene used in this study, the dominating degradation reaction is the addition of radicals to the vinyl group located at the end of each polymer chain. Usually, a strong correlation exists between the consumption of vinyl groups and the change of viscosity during the processing of the polymer. The correlation between vinyl content and the MFR of the polymer is presented in Figure 10 for all the additive packages. The correlation is indeed very close, showing that the general trend is valid also for natural antioxidants. Stabilizer packages containing a phenolic, primary stabilizer and secondary antioxidant are very efficient processing stabilizers; they prevent the reaction of the vinyl group and the change of melt flow rate. The situation is completely different for residual stability. Although the extracts of grape peel are more efficient processing stabilizers than *trans*-resveratrol or the commercial stabilizer, I1010, they do not offer practically any or only very small residual stability. The OIT of the PE compounds after the first extrusion is plotted against the change in MFR across the six extrusion steps in Figure 11. According to the figure, melt stabilization efficiency is inversely proportional to the long-term stabilization effect of the studied additives. Although we do not claim that the correlation is universal, it is worth contemplating the reason for the inverse correlation between the two characteristics.

One of the reasons is certainly the dissimilar chemical structure of the active compounds in the extracts and that of the two compounds used as references. The extracts contain considerable amounts of polyphenols with complicated multiple-ring structures [23,41]. Although quite a few phenolic OH groups are located on the rings, which have active hydrogens capable of reacting with radicals, the rate of reaction might be different with different types of radicals [43]. Hindered phenols are known to react preferably with oxygen-centered radicals. Processing stability, on the other hand, mainly depends on the reaction of alkyl radicals and their scavenging by the stabilizer. Polyphenols obviously and readily react with alkyl radicals but not with oxygen-centered ones, while the opposite is valid for hindered phenols and for *trans*-resveratrol. Although this tentative explanation is plausible, it definitely needs further verification. The low inherent stability of the natural extracts must be mentioned as well. Strangely, decomposition, probably because of the glycoside structure of the compounds, does not hinder melt stabilization, but it might affect long-term stability in oxygen excess.

Finally, another interesting observation must be mentioned here. The DPPH assay is widely used for the characterization of the antioxidant activity of a wide range of compounds. The assay and the IC50 value seem to have very limited value in the assessment of the stabilization efficiency of compounds used for the stabilization of polymers and definitely not in estimating residual stability. According to Table 2, the IC50 of I1010 is quite large compared with the other studied compounds, but it offers a good overall stabilization effect both in processing and for residual stability. The polymer containing *trans*-resveratrol with a smaller IC50 value has the best residual stability but poorer processing stability than with I1010 and especially with the extracts. These latter, with a comparable value to *trans*-resveratrol, offer negligible long-term stabilization for the polymer. Obviously, the IC50 value cannot be used for the estimation of the stabilization efficiency of potential additives, including natural antioxidants and their extracts.

## 4. Materials and Methods

### 4.1. Materials

The polymer used in the experiments was the Tipelin FS 471 grade ethylene/1-hexene copolymer (melt flow rate: 0.3 g/10 min at 190 °C and 2.16 kg; nominal density: 0.947 g/cm^3^) polymerized with a Phillips catalyst. The additive-free polymer powder was provided by the MOL Group Ltd. (Tiszaújváros, Hungary). The *trans*-resveratrol (99%) was purchased from CurEase (McEwen, Flanklin, TN, USA), and the quercetin (99%) standard was obtained from Merck KGaA (Darmstadt, Germany) for the quantitative determination of these components in the extract and for the stabilization experiments. HPLC gradient grade solvents (acetonitrile, ethanol) purchased from Merck KGaA (Darmstadt, Germany) were used for quantitative analysis. 2,2-diphenyl-1-picrylhydrazyl (DPPH) was acquired from Merck for the DPPH assay. Folin–Ciocalteu phenol reagent (2 N) and sodium carbonate (anhydrous, ACS reagent >99.5%) were also obtained from Merck. The pyrogallol (>98% HPLC) standard used for the measurement of total polyphenol content was purchased from Honeywell-Fluka (Charlotte, NC, USA). Both the 96% ethanol and methanol were obtained from Molar Chemicals Ltd. (Halásztelek, Hungary).

### 4.2. Extraction

The extracts used for stabilization were prepared from dried red grape peel powder obtained from the Gere Winery (Villány, Hungary). The powder was extracted with supercritical CO_2_ fluid (SFE) containing 10 wt% ethanol at 300 bar and 40 °C with a single separator (operated at 40 bar and 40 °C). An oily green extract with 11.5% yield (g extract/100 g dry grape peel) was obtained in the procedure, mainly coming from grape seed contamination of the peel.

Three different methods were used to extract polyphenols from the SFE residue obtained in the previous stage. Soxhlet extraction was carried out with 96% EtOH until the complete exhaustion of the powder (approximately 48 h). Ethanol was removed by vacuum distillation. A total of 1.62 g purple crystals were obtained with a yield of 8.3 wt%.

Active components were also extracted by simple stirring in a tank reactor using 96% ethanol. The residue-to-solvent ratio was 1:6 mass to volume. A total of 100.8 g of SFE residue was placed in a 2 L round-bottom flask, and then 600 mL of 96% EtOH was added. The solution was stirred at 405–425 rpm for 3 h at 40 °C. Subsequently, the solution was filtered using a vacuum jet valve and filter paper. After the evaporation of the solvent from the solution, dark purple crystals were obtained with 3.7% yield. Extraction was repeated once more, and the obtained extracts were combined into one batch.

Polyphenols were also extracted by ultrasonic treatment using a Hielscher UP200St device. Extraction was carried out with EtOH at a 1:20 residue-to-solvent ratio at 40 °C. Three consecutive extractions were performed for 10 min by adding fresh solvent at each step. A total of 10.19 g of SFE residue and 200 mL of 96% EtOH were placed into a 500 mL glass flask covered with aluminum foil to prevent light degradation. The extracts obtained in the three extraction steps were combined, resulting in 0.50 g dark-purple crystal-like material with a yield of 5.10%.

### 4.3. Characterization

The components of the extracts were analyzed with LC-MS/MS. Relevant components were identified by their characteristic MRM (multiple-reaction monitoring) transitions reported in the literature [23], while the amount of *trans*-resveratrol and quercetin was quantified. A total of 10 mg of grape peel extract was weighed into a 10 mL volumetric flask and then dissolved in methanol. A Shimadzu NexeraX2 system (Kyoto, Japan) coupled with a Shimadzu LC-MS-8060 triple quadrupole tandem mass spectrometer (Kyoto, Japan) was used for the analytical measurements. Precisely 5 µL of the processed sample was injected into the chromatograph equipped with an Inertsil ODS-4 column (2.1 × 75 mm, 3 µm, GL Sciences Inc., Japan) with a guard column (1.5 × 10 mm) of the same stationary phase tempered at 25 °C. Gradient elution was applied with mobile phases A: 1% formic acid and B: acetonitrile at a flow rate of 0.5 mL/min. The initial eluent composition was 10% B for 1.0 min. Then, a linear gradient was applied to 50% B until 8.0 min post-injection. Afterward, a linear gradient was applied to 90% B until 9.0 min post-injection. That composition was kept for 2.0 min, and then the column was re-equilibrated to the initial condition until the stop time of 13.0 min. The mass spectrometric parameter settings were as follows: MRM detection mode, electrospray ionization in the negative mode, interface temperature at 300 °C, desolvation line temperature at 200 °C and interface voltage of −3 kV. Quercetin was quantified at the transition of *m*/*z* 301.00 → 151.15 with the collision energy of 22.0 V, and a qualifier ion was set at the transition of *m*/*z* 301.00 → 179.15 with the collision energy of 19.0 V and 50 ms of dwell time. The retention time of quercetin was 6.0 min. *Trans*-resveratrol was quantified at the transition of *m*/*z* 227.10 → 143.20 with the collision energy of 24.0 V, and a qualifier ion was set at the transition of *m*/*z* 227.10 → 185.15 with the collision energy of 18.0 V and 50 ms of dwell time. The retention time of *trans*-resveratrol was 5.6 min. Based on the literature data [23], MRM transitions of further compounds were set for monitoring, but due to the lack of standards, they were not quantified.

The antioxidant capacity of the extracts, resveratrol and I1010 was determined by the DPPH method [44] using the 1,1-diphenyl-2-picrylhydrazyl (DPPH) free radical and a Camspec M501 (Leeds, UK) spectrophotometer. Three parallel measurements were carried out for each concentration in methanol. The activity of the studied compounds was characterized by the IC50 value, which is the concentration of the substrate that causes a 50% loss of DPPH activity. A smaller IC50 value indicates comparatively larger activity. Total polyphenol content was measured by spectrophotometry using pyrogallol as a reference. The measurement was carried out with a Camspec M501 spectrophotometer [45]. Folin reagent was used, and the absorbance at 760 nm indicates polyphenol content.

The possible crystalline structure of the extract and the reference compounds was studied by differential scanning calorimetry (DSC). The equipment used was a Perkin Elmer DSC 7 (Norwalk, CT, USA) apparatus. The measurements were performed on 3–5 mg samples, which were heated from 30 to 300 °C at a 10 °C/min heating rate in oxygen. The inherent stability and the decomposition of the studied additives were investigated by thermogravimetric analysis (TGA). A Perkin Elmer TGA 6 (Norwalk, CT, USA) apparatus was used for the measurements. Samples of 3–5 mg weight were heated from 30 to 700 °C at a 10 °C/min heating rate in oxygen.

### 4.4. Sample Preparation

The additives and the polymer powder were homogenized first in a Henschel FM/A10 high-speed mixer (Thyssen, Kassel, Germany) at the mixing rate of 1000 rpm for 10 min. The extracted compounds and *trans*-resveratrol were added to the powder in solution (ethanol, 96%) to achieve uniform distribution. Ethanol was evaporated at ambient temperature. The produced dry blend was extruded and pelletized in six consecutive steps at 50 rpm. The barrel temperatures of the extruder were set to 180, 220, 260 and 260 °C. The extruder used was a Rheomex S ¾″ (Haake, Saddle Brook, NJ, USA) type single screw extruder driven by a Haake Rheocord EU 10 V (Haake, Saddle Brook, NJ, USA) unit. Pellets were collected after each extrusion step. Films of about 100 µm thickness were compression molded from the pellets for further studies. Compression molding was carried out at 190 °C and 5 min (3 min preheating and 2 min compression time) using a Fontijne SRA 100 (Vlaardingen, The Netherlands) machine.

The amount of the grape peel extracts and *trans*-resveratrol was 1000 ppm in the polymer, and 1000 ppm Sandostab PEPQ (PEPQ, Clariant, Basel, Switzerland) secondary stabilizer was added to all antioxidant formulations. A compound containing only 1000 ppm PEPQ alone was also prepared and studied for comparison. A polymer sample stabilized with 1000 ppm Irganox 1010 (I1010, BASF, Nienburg/Weser, Germany) and 1000 ppm PEPQ was produced for comparative purposes as well. The neat polyethylene powder alone, without any additives, was also studied as a reference.

### 4.5. Measurements

The chemical structure of polyethylene was studied by Fourier-transform infrared spectroscopy (FTIR) in transmission mode on the films of 100 μm thickness prepared by compression molding. The equipment used for the purpose was a Tensor 27 (Bruker Optik GmbH, Leipzig, Germany) spectrophotometer. Five spectra were recorded on each sample between 4000 and 400 cm^−1^ wavenumbers at 2 cm^−1^ resolution with 16 scans. The number of vinyl groups per 1000 carbon atoms was calculated from the intensity of the absorption band appearing at 908 cm^−1^. The details of the determination of the concentration of unsaturated groups are described in one of our previous publications [21]. Changes in the rheological characteristics of the polymer were followed by the measurement of melt flow rate (MFR). The tests were performed according to the ASTM D 1238-79 standard at 190 °C and 2.16 kg load using a Göttfert MPS-D (Buchen, Germany) MFR apparatus. The MFR of each sample was measured five times. The amount of residual antioxidants was estimated by the measurement of the oxidation induction time (OIT) using a Perkin Elmer DSC 7 (Norwalk, CT, USA) apparatus. The experiments were carried out using open aluminum pans in oxygen atmosphere at a constant, 20 mL/min flow rate of oxygen and 190 °C. The measurements were performed in triplicates according to the ISO 11357-6:2018 standard. The color of the samples was characterized by the whiteness index (WI) using a Hunterlab ColorQuest 45/0 (Reston, VA, USA) apparatus. The measurements were performed in triplicates.

## 5. Conclusions

The extraction of dry grape peel by three different techniques, stirred tank reactor, Soxhlet and ultrasound extraction, produced similar amounts of extracts. The composition of the extracts was also similar, only the exact amount of the various components differed somewhat. Ten major compounds were identified in the extracts, which contained a considerable number of polyphenols, but contrary to results published in the literature, only a small amount of quercetin and a negligible quantity of *trans*-resveratrol. The characteristics of the three extracts were similar independently of the extraction technology. The extracts proved to be more efficient processing stabilizers than *trans*-resveratrol and the commercial stabilizer used in the largest quantity in industrial practice, irrespective of the extraction technology used. Since the extracts do not contain much quercetin and *trans*-resveratrol, stabilization must be attributed to other polyphenols. In spite of the good processing stabilization effect, polymers containing the extracts had very poor residual stability. The dissimilarities in processing and long-term stabilization must be related to the different structures of the polyphenols in the extracts and the reference compounds. The results clearly proved that the IC50 value determined by the DPPH assay is not suitable for the estimation of the efficiency of a compound as a stabilizer for polymers.

## Figures and Tables

**Table 1:** Composition of the grape peel extracts produced by various extraction techniques.

Technique	Yield (wt%)	Polyphenol ^1^ (wt%)	*Trans*-resveratrol (wt%)	Quercetin (wt%)
Stir	3.68	16.3	0.0313	0.459
Sox	8.31	16.5	0.0044	0.401
USound	5.10	11.5	0.0178	0.371

^1^ g pyrogallol eq/100 g extract.

**Table 2:** Antioxidant activity and polyphenol content of the grape peel extracts produced, some of their components and the hindered phenolic antioxidant (I1010) used as reference.

Component	IC50 ^1^ (μg/mL)	Polyphenol (wt%)
Irganox 1010	129.3	–
*Trans*-resveratrol	21.7	–
Quercetin	4.5	–
StirExt	17.7	16.3
SoxExt	18.3	16.5
USoundExt	14.3	11.5

^1^ Determined by the DPPH assay.

## Data Availability

Not applicable.

## Sample Availability

Samples of the compounds are available upon request from the authors.

## References

[1] Zweifel H. (1998). Stabilization of Polymeric Materials.

[2] Rideal G.R., Padget J.C. (1976). The thermal-mechanical degradation of high density polyethylene. J. Polym. Sci. Polym. Symp..

[3] Allen N.S., Edge M. (2021). Perspectives on additives for polymers. 1. Aspects of stabilization. J. Vinyl Addit. Technol..

[4] Kriston I., Orbán-Mester A., Nagy G., Staniek P., Földes E., Pukánszky B. (2009). Melt stabilisation of Phillips type polyethylene, Part I: The role of phenolic and phosphorous antioxidants. Polym. Degrad. Stab..

[5] Brocca D., Arvin E., Mosbæk H. (2002). Identification of organic compounds migrating from polyethylene pipelines into drinking water. Water Res..

[6] Kirschweng B., Tátraaljai D., Földes E., Pukánszky B. (2017). Natural antioxidants as stabilizers for polymers. Polym. Degrad. Stab..

[7] Azmir J., Zaidul I.S.M., Rahman M.M., Sharif K.M., Mohamed A., Sahena F., Jahurul M.H.A., Ghafoor K., Norulaini N.A.N., Omar A.K.M. (2013). Techniques for extraction of bioactive compounds from plant materials: A review. J. Food Eng..

[8] Pietta P.-G. (2000). Flavonoids as antioxidants. J. Nat. Prod..

[9] Gregorová A., Cibulková Z., Košíková B., Šimon P. (2005). Stabilization effect of lignin in polypropylene and recycled polypropylene. Polym. Degrad. Stab..

[10] Kun D., Pukánszky B. (2017). Polymer/lignin blends: Interactions, properties, applications. Eur. Polym. J..

[11] Kosikova B., Kacurakova M., Demianova V. (1993). Photooxidation of the composite lignin/polypropylene films. Chem. Pap..

[12] Al-Malaika S., Ashley H., Issenhuth S. (1994). The antioxidant role of α-tocopherol in polymers. I. The nature of transformation products of α-tocopherol formed during melt processing of LDPE. J. Polym. Sci. A Polym. Chem..

[13] Al-Malaika S., Goodwin C., Issenhuth S., Burdick D. (1999). The antioxidant role of α-tocopherol in polymers II. Melt stabilising effect in polypropylene. Polym. Degrad. Stab..

[14] Al-Malaika S., Issenhuth S. (1999). Antioxidant role of α-tocopherol in polymers. III. Nature of transformation products during polyolefins extrusion. Polym. Degrad. Stab..

[15] Tátraaljai D., Földes E., Pukánszky B. (2014). Efficient melt stabilization of polyethylene with quercetin, a flavonoid type natural antioxidant. Polym. Degrad. Stab..

[16] Samper M., Fages E., Fenollar O., Boronat T., Balart R. (2013). The potential of flavonoids as natural antioxidants and UV light stabilizers for polypropylene. J. Appl. Polym. Sci..

[17] Morici E., Arrigo R., Dintcheva N.T. (2014). Quercetin as natural stabilizing agent for bio-polymer. AIP Conference Proceedings.

[18] Kirschweng B., Tilinger D.M., Hégely B., Samu G., Tátraaljai D., Földes E., Pukánszky B. (2018). Melt stabilization of PE with natural antioxidants: Comparison of rutin and quercetin. Eur. Polym. J..

[19] Kirschweng B., Bencze K., Sárközi M., Hégely B., Samu G., Hári J., Tátraaljai D., Földes E., Kállay M., Pukánszky B. (2016). Melt stabilization of polyethylene with dihydromyricetin, a natural antioxidant. Polym. Degrad. Stab..

[20] Kirschweng B., Vörös B., Arroussi M., Tátraaljai D., Zsuga M., Pukánszky B. (2021). Melt stabilization of polyethylene with natural antioxidants: Comparison of a natural extract and its main component. J. Therm. Anal. Calorim..

[21] Tátraaljai D., Tang Y., Pregi E., Vági E., Pukánszky B. (2022). Pomegranate extract for the processing stabilization of polyethylene. J. Vinyl Addit. Technol..

[22] Tátraaljai D., Tang Y., Pregi E., Vági E., Horváth V., Pukánszky B. (2022). Stabilization of PE with Pomegranate Extract: Contradictions and Possible Mechanisms. Antioxidants.

[23] Milinčić D.D., Stanisavljević N.S., Kostić A.Ž., Soković Bajić S., Kojić M.O., Gašić U.M., Barać M.B., Stanojević S.P., Lj Tešić Ž., Pešić M.B. (2021). Phenolic compounds and biopotential of grape pomace extracts from Prokupac red grape variety. LWT.

[24] Jackson R.S. (1994). Wine Science: Principles and Applications.

[25] Pinelo M., Rubilar M., Jerez M., Sineiro J., Núñez M.J. (2005). Effect of solvent, temperature, and solvent-to-solid ratio on the total phenolic content and antiradical activity of extracts from different components of grape pomace. J. Agric. Food Chem..

[26] Pinelo M., Arnous A., Meyer A.S. (2006). Upgrading of grape skins: Significance of plant cell-wall structural components and extraction techniques for phenol release. Trends Food Sci. Technol..

[27] Cantos E., Espin J.C., Tomás-Barberán F.A. (2002). Varietal differences among the polyphenol profiles of seven table grape cultivars studied by LC–DAD–MS–MS. J. Agric. Food Chem..

[28] Zhu L., Zhang Y., Lu J. (2012). Phenolic contents and compositions in skins of red wine grape cultivars among various genetic backgrounds and originations. Int. J. Mol. Sci..

[29] Du Y., Li X., Xiong X., Cai X., Ren X., Kong Q. (2021). An investigation on polyphenol composition and content in skin of grape (*Vitis vinifera* L. cv. Hutai No. 8) fruit during ripening by UHPLC-MS2 technology combined with multivariate statistical analysis. Food Biosci..

[30] Jeandet P., Bessis R., Gautheron B. (1991). The production of resveratrol (3,5,4′-trihydroxystilbene) by grape berries in different developmental stages. AJEV.

[31] Cerruti P., Malinconico M., Rychly J., Matisova-Rychla L., Carfagna C. (2009). Effect of natural antioxidants on the stability of polypropylene films. Polym. Degrad. Stab..

[32] Ambrogi V., Cerruti P., Carfagna C., Malinconico M., Marturano V., Perrotti M., Persico P. (2011). Natural antioxidants for polypropylene stabilization. Polym. Degrad. Stab..

[33] Nanni A., Messori M. (2018). A comparative study of different winemaking by-products derived additives on oxidation stability, mechanical and thermal proprieties of polypropylene. Polym. Degrad. Stab..

[34] Cerruti P., Santagata G., Gomez d’Ayala G., Ambrogi V., Carfagna C., Malinconico M., Persico P. (2011). Effect of a natural polyphenolic extract on the properties of a biodegradable starch-based polymer. Polym. Degrad. Stab..

[35] Persico P., Ambrogi V., Baroni A., Santagata G., Carfagna C., Malinconico M., Cerruti P. (2012). Enhancement of poly(3-hydroxybutyrate) thermal and processing stability using a bio-waste derived additive. Int. J. Biol. Macromol..

[36] Nanni A., Ricci A., Versari A., Messori M. (2020). Wine derived additives as poly(butylene succinate) (PBS) natural stabilizers for different degradative environments. Polym. Degrad. Stab..

[37] Busolo M.A., Lagaron J.M. (2015). Antioxidant polyethylene films based on a resveratrol containing clay of interest in food packaging applications. Food Packag. Shelf Life.

[38] Gülçin İ. (2010). Antioxidant properties of resveratrol: A structure–activity insight. IFSET.

[39] Latruffe N., Lançon A., Limagne E., Michaille J.J., Claus Jacob G.K., Slusarenko A., Winyard P.G., Burkholz T. (2014). Bioreactivity of resveratrol toward inflammation processes. Recent Advances in Redox Active Plant and Microbial Products: From Basic Chemistry to Widespread Applications in Medicine and Agriculture.

[40] Latruffe N., Vervandier-Fasseur D. (2018). Strategic Syntheses of Vine and Wine Resveratrol Derivatives to Explore Their Effects on Cell Functions and Dysfunctions. Diseases.

[41] Georgiev V., Ananga A., Tsolova V. (2014). Recent advances and uses of grape flavonoids as nutraceuticals. Nutrients.

[42] Allen N.S., Edge M., Hussain S. (2022). Perspectives on Yellowing in the Degradation of Polymer Materials: Inter-relationship of Structure, Mechanisms and Modes of Stabilisation. Polym. Degrad. Stab..

[43] Cai W., Chen Y., Xie L., Zhang H., Hou C. (2014). Characterization and density functional theory study of the antioxidant activity of quercetin and its sugar-containing analogues. Eur. Food Res. Technol..

[44] Molyneux P. (2004). The use of the stable free radical diphenylpicrylhydrazyl (DPPH) for estimating antioxidant activity. Songklanakarin J. Sci. Technol..

[45] Singleton V.L., Orthofer R., Lamuela-Raventós R.M. (1999). Analysis of total phenols and other oxidation substrates and antioxidants by means of folin-ciocalteu reagent. Methods Enzymology.

